# Origin of Positive Aging in Quantum‐Dot Light‐Emitting Diodes

**DOI:** 10.1002/advs.201800549

**Published:** 2018-07-03

**Authors:** Qiang Su, Yizhe Sun, Heng Zhang, Shuming Chen

**Affiliations:** ^1^ Department of Electrical and Electronic Engineering Southern University of Science and Technology Shenzhen 518055 P. R. China

**Keywords:** exciton quenching, interfacial reaction, light‐emitting diodes, positive aging, quantum‐dots

## Abstract

The phenomenon of positive aging, i.e., efficiency increased with time, is observed in quantum‐dot light‐emitting diodes (QLEDs). For example, the external quantum efficiency (EQE) of blue QLEDs is significantly improved from 4.93% to 12.97% after storage for 8 d. The origin of such positive aging is thoroughly investigated. The finding indicates that the interfacial reaction between Al cathode and ZnMgO electron transport layer accounts for such improvement. During shelf‐aging, the Al slowly reacts with the oxygen from ZnMgO, and consequently, leads to the formation of AlO*_x_* and the production of oxygen vacancies in ZnMgO. The AlO*_x_* interlayer reduces the electron injection barrier while the oxygen vacancies increase the conductivity of ZnMgO and, as a result, the electron injection is effectively enhanced. Moreover, the AlO*_x_* can effectively suppress the quenching of excitons by metal electrode. Due to the enhancement of electron injection and suppression of exciton quenching, the aged blue, green, and red QLEDs exhibit a 2.6‐, 1.3‐, and 1.25‐fold efficiency improvement, respectively. The studies disclose the origin of positive aging and provide a new insight into the exciton quenching mechanisms, which would be useful for further constructing efficient QLED devices.

Light‐emitting diodes (LEDs) based on colloidal quantum dots (QDs) have received considerable research attention recently due to their excellent optoelectronic properties such as saturated and tunable emission colors, high efficiency, and low‐cost solution processability, which make them promising candidates for next generation display.^1^, ^2^, ^3^, ^4^, ^5^, ^6^ After two decades of rapid development, the external quantum efficiencies (EQEs) of quantum‐dot LEDs (QLEDs) have been substantially improved from 0.01%^7^ to over 20%.^8^ Very recently, by using tandem structures, the EQEs of blue, green, red QLEDs have further been improved to 21.4%, 27.6%, and 23.1%,^9^ respectively. Currently, the efficiencies of state‐of‐the‐art QLEDs are very close to those of organic LEDs (OLEDs), while the color saturation and the fabrication cost are much better than those of OLEDs, and thus QLEDs are expected to be the successors of OLEDs for next generation wide‐color‐gamut printing display application.

Though QLEDs and OLEDs share lots of similarities such as device structures, fabrication, and characterization processes, the working mechanisms including charge injection, charge recombination, and exciton quenching, are quite different. For example, in typical QLEDs, electron injection is relatively easy due to small injection barrier and high electron mobility of ZnO electron transport layer (ETL),^10^ whereas in most OLEDs, electron injection is relatively inefficient.^11^, ^12^, ^13^ In addition, in inorganic QDs, the electrons and the holes are bounded by a weak Coulombic attraction, leading to the formation of Wannier‐type excitons,^14^ whereas in organic emitters, the excitons are Frenkel‐type which have relatively strong binding energy.^15^ The difference of bounding force may result in different quenching pathways. For example, due to the weak binding energy, the QD excitons are easily dissociated by adjacent metal oxide^10^, ^16^ or can nonradiatively relax through Auger recombination.^17^, ^18^, ^19^ Very recently, Acharya et al. observed that the efficiency and the luminance of QLEDs are significantly enhanced after shelf‐aging for several days and they termed this phenomenon as positive aging.^20^ Such abnormal phenomenon has never been observed in OLEDs, which implies that the degradation mechanism of QLEDs is rather different with that of OLEDs. Acharya et al. concluded that the positive aging is caused by the encapsulation resin, which reacts with ZnO and consequently leads to the formation of carbonate that helps to reduce ZnO defect densities.^20^ However, the exact origin of positive aging still remains unclear.

In this contribution, the origin of positive aging is thoroughly investigated. We observed that at a certain driving voltage, the aged devices exhibit larger current density and higher EQE. To identify critical factors accounting for the improved efficiency, the effects of encapsulation resins, cathode materials, and ETL materials on the aging behavior are systematically studied. We identify that the interfacial reaction between Al cathode and ZnMgO ETL plays a major role in positive aging, which is rather different from the conclusions of Acharya et al. During shelf‐aging, the Al slowly reacts with the oxygen from ZnMgO, and consequently, leads to the formation of AlO*_x_* and the production of oxygen vacancies in ZnMgO. The AlO*_x_* interlayer reduces the electron injection barrier while the oxygen vacancies increase the electron concentration of ZnMgO, and as a result, the aged devices exhibit larger electron current. Moreover, the AlO*_x_* can effectively suppress the quenching of excitons by metal electrode. Due to enhancement of electron injection and suppression of exciton quenching, the 8 d aged blue, green, and red QLEDs exhibit high EQEs of 12.97%, 15.34%, and 18.53%, which are about 2.6‐, 1.3‐, and 1.25‐fold higher than 4.93%, 11.62%, and 15.30% of the fresh blue, green, and red devices, respectively. Our studies disclose the origin of positive aging and may provide a new insight into the exciton quenching mechanisms, which would be useful for further constructing efficient QLED devices.

Blue, green, and red QLEDs with structure of glass/ITO/PEDOT:PSS/TFB/QDs/ZnMgO/Al were fabricated and characterized. The schematic structure and the energy band diagram are shown in **Figure** **1**. To relieve strain caused by lattice mismatch, alloyed QDs based on Cd*_x_*Zn_1−_ *_x_*Se@ZnS and Cd*_x_*Zn_1−_ *_x_*Se*_y_*S_1−_ *_y_*@ZnS are used, which are widely adopted to make efficient devices.^17^, ^21^, ^22^, ^23^ To exclude the influence of oxygen and moisture on aging behavior, the devices were stored in N_2_‐filled glove box after fabrication. To evaluate the effect of shelf‐aging on the device characteristics, the devices were tested immediately after fabrication and after storage for 3, 5, and 8 d. There are four devices on each patterned ITO substrate, and to exclude the influence of electrical stress, a new device (i.e., untested before) was tested at each testing session. The device characteristics of the blue, green, and red QLEDs tested at different time period are shown in **Figure** **2**a–f. From the data shown in Figure 2a–f, it is clear that: 1) at a certain driving voltage, the current density is significantly increased if the devices were shelf‐aged for several days; 2) the luminance is gradually enhanced with increasing of shelf‐aging time, and 3) the EQE is gradually enhanced; for example, for the blue QLEDs, the EQE is substantially enhanced from 4.93% to 8.23% after storage for 3 d and is further boosted to 12.97% after storage for 8 d. The improvement is quite significant in the initial several days; however, with time prolonging, the increment rate becomes slow and after 8 d aging, only slight improvement is observed if the storage time is further increased. Similar phenomena are also observed in red and green devices, whose EQEs are effectively improved from 11.62% and 15.30% to 15.34% and 18.53%, respectively. The blue QLEDs exhibit a 2.6‐fold efficiency improvement after 8 d aging, which is the highest compared to 1.3‐ and 1.25‐fold of the green and the red devices, respectively. Figure 2g shows the electroluminescence (EL) spectra of the devices. Figure 2h shows the photos of the devices and Figure 2i shows the magnified images which were taken through the eye‐lens of PR670 spectroscopy. Very uniform emission can be observed, indicating the high uniformity of the solution‐deposited film, and thus excludes the influence of film's defects on the aging behavior. The detailed performance data are summarized in **Table** **1**. When the devices are under accelerated aging, i.e., stressed by a continuous constant current, the phenomenon of positive aging can also be observed, as shown in Figure S1 (Supporting Information). The luminance is gradually increased over a long period of time and after reaching the maximum value, the luminance starts to drop, which is consistent with the behavior of shelf‐aging. However, the electrical aging behavior is more complex than that of the shelf‐aging, and further detailed investigation is needed. It should be noted that the phenomenon of positive aging can only be observed in QDs with high stability. If the QDs have low reliability, the intrinsic degradation of QDs is dominant, and in this scenario, negative aging behavior is observed.

**Figure 1:**
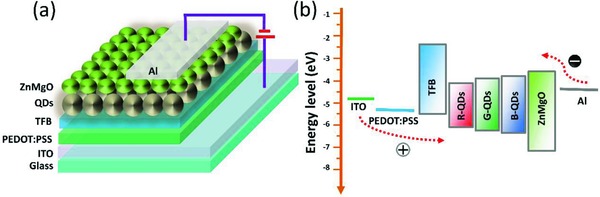
a) Device structure and b) energy band diagram of the QLEDs.

**Figure 2:**
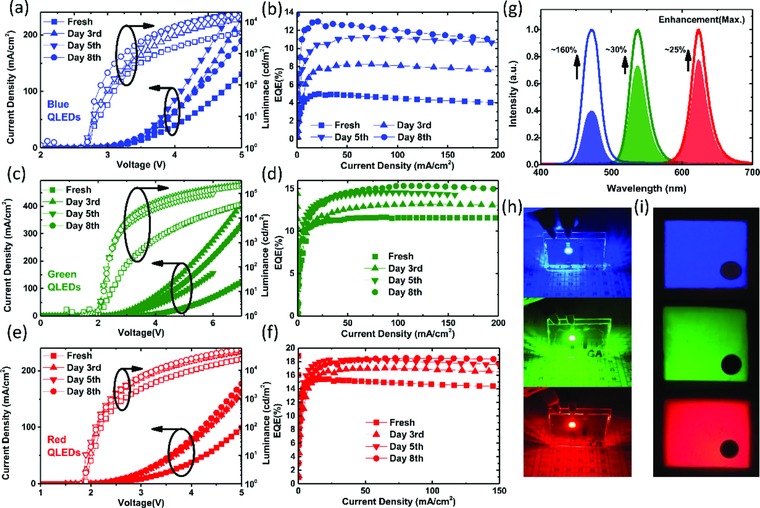
a,c,e) *J*–*V*–*L* characteristics. b,d,f) EQE‐J characteristics of devices tested at different time period. g) EL spectra, h) photos of the devices, and i) magnified images of the devices taken through the eye‐lens of PR 670 spectroscopy.

**Table 1:** Key data of device performance

Devices		Peak [FWHM]	*V*_on_ [V] [1 cd cm^−2^]	Luminance [cd m^−2^]	CE [cd A^−1^]	EQE [%]	PE [lm W^−1^]	EQE Enhancement [%]
				5 [V]	Max	Max	Max	
Blue	Fresh	470 nm	2.6	4459	3.83	4.93	3.36	≈160
QLED	Aged	(25 nm)	2.6	18 348	11.38	12.97	10.42	
Green	Fresh	537 nm	2.1	5621	52.06	11.62	34.73	≈30
QLED	Aged	(28 nm)	2.1	83 480	68.76	15.34	45.85	
Red	Fresh	624 nm	1.8	24 644	25.86	15.30	21.37	≈25
QLED	Aged	(25 nm)	1.8	54 669	31.36	18.53	24.01	

The positive aging is likely due to: 1) the interaction between encapsulation resin and ZnO as reported by Acharya et al.^20^; 2) the improvement of QD photoluminescence (PL) quantum yield, and 3) the reduction of ZnO defect densities. To find out the exact origin of positive aging, the effects of encapsulation resins, cathode materials, and ETL materials on the aging behavior are systematically investigated. **Figure** **3**a shows the EQE‐J characteristics of the blue QLEDs with and without encapsulation. After 5 d aging, the devices exhibit higher EQE and larger current density. The EQE improvement of the unencapsulated device is even more significant than that of the encapsulated device, indicating that the encapsulation resin does not play the major role in positive aging. We have tested three kinds of encapsulation resins, and the results are quite similar. After excluding the influence of encapsulation, the effects of QDs and ZnO on aging behavior are then investigated. To rule out the influence of cathode, one of the devices without Al cathode was fabricated and kept with other samples in glove box. On the fifth day, the Al cathode was deposited and the device was characterized soon after the deposition. As shown in Figure 3b, device with Al cathode deposited on the fifth day does not exhibit positive aging; the EQE is decreased from 5.9% to 3.7% while the current density is almost the same, indicating that the QDs and the ZnMgO are not the dominant factors responsible for the positive aging. However, for the device with Al cathode deposited at the very beginning, positive aging is observed. Similar phenomena are also observed in red and green devices, as shown in Figure S2 (Supporting Information). It seems that the Al cathode can affect the aging behavior. Inverted device with structure of glass/ITO/ZnMgO/QDs/TcTa/NPB/HATCN/Al was also fabricated and characterized. As shown in Figure 3c and Figure S3 (Supporting Information), no positive aging effect is observed for the inverted device, in which the Al cathode is contacted with the organic hole injection layer. Therefore, through comparison, we can boldly predict that the positive aging is largely affected by the interface of Al/ZnMgO. The influence of the cathode materials, i.e., Ag, Cu, Al, and ITO, on the aging behavior is further investigated. As shown in Figure 3d–f, device with ITO cathode does not exhibit positive aging, while it is observed for devices with metal cathode Al, Ag, and Cu, respectively. Device with Al exhibits the most significant efficiency improvement, while there is only slight improvement for the device with Ag cathode. Among the cathode materials under investigation, the degree of chemical reactivity is Al > Cu > Ag > ITO. The efficiency enhancement is closely related with chemical reactivity; we thus speculate that it is the interaction between metal electrode and ZnMgO that accounts for the positive aging.

**Figure 3:**
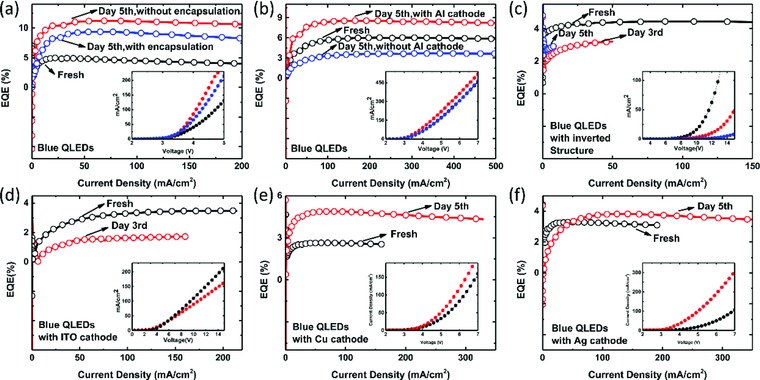
EQE‐*J* characteristics and *J*–*V* characteristics (inset) of blue QLEDs tested at different time period a) with and without encapsulation, b) with and without Al cathode at the very beginning, c) with inverted structure, d–f) with different cathode materials.

The interaction between Al and ZnMgO is further investigated. The Al can be easily oxidized by oxygen from ambience due to its high chemical reactivity.^24^ The Al cathode is closely contacted with the ZnMgO ETL, and thus during shelf‐aging, the Al may slowly react with the oxygen from the ZnMgO, leading to the formation of AlO*_x_* and the accumulation of oxygen vacancies at the surface of ZnMgO.^25^, ^26^, ^27^ To verify our hypothesis, the chemical bonds of Al and oxygen at the interface of Al/ZnMgO were characterized by X‐ray photoelectron spectroscopy (XPS). To precisely probe the interface of Al/ZnMgO, the Al was removed by sputter etching and the etching depth was monitored in situ by analyzing the amountelements. As shown in **Figure** **4**a, the O 1s spectra of ZnMgO exhibit two peaks, which are centered at 529.6 ± 0.1 eV (O‐1) and 531.1 ± 0.1 eV (O‐2), respectively. The O‐1 is related to the oxygen in oxide lattices, and the O‐2 consists of oxygen vacancies and oxygen bonds in the hydroxide. It is clear that after 5 d aging, the intensity of O‐2 is higher than that of the fresh sample. This is because the oxygen is out‐diffused from the ZnMgO layer and subsequently reacts with the Al, leading to the accumulation of oxygen vacancies at the interface of Al/ZnMgO.^25^, ^26^, ^27^ The formation of interfacial AlO*_x_* can be verified from the Al 2p spectra shown in Figure 4b. The Al 2p spectra consist of two peaks, which are centered at 72.6 ± 0.1 eV and 75.3 ± 0.1 eV, corresponding to Al and amorphous AlO*_x_*, respectively. Obviously, after 5 d aging, the intensity of AlO*_x_* is higher than that of the fresh sample. In this way, an AlZnMgO alloyed interface would be formed. In addition, the interfacial AlO*_x_* can significantly increase the electron injection by reducing the injection barrier.^28^, ^29^ Moreover, the accumulation of oxygen vacancies at the interface can increase the electron concentration of ZnMgO, as oxygen vacancies are reported to act as donors of ZnMgO.^30^, ^31^ The alloyed interface, the formation of AlO*_x_*, and the improvement of ZnMgO conductivity all contribute to the improvement of electron injection, and thus the aged devices exhibit significantly larger current density, as shown in Figure 2. This also can be verified by measuring the electron current in electron‐only device. As shown in Figure 4c, the aged device indeed exhibits larger electron current. It should be noted that, in blue QLEDs, the electron and the hole injection are relatively inefficient due to the wide bandgap of the blue QDs. Figure 4d shows the hole current in the hole‐only device, which is almost unchanged even after aging for 5 d. For the fresh sample, the hole current is larger than the electron current. After 5 d aging, the electron current is significantly enhanced, which thus improves the charge balance and consequently enhances the efficiency of the blue QLEDs. However, in other color QLEDs, especially the red QLEDs, the electron injection is very efficient,^8^, ^32^ and thus the enhancement of electron injection cannot improve the efficiency of red and green QLEDs, which implies that there are other mechanisms responsible for performance improvement.

**Figure 4:**
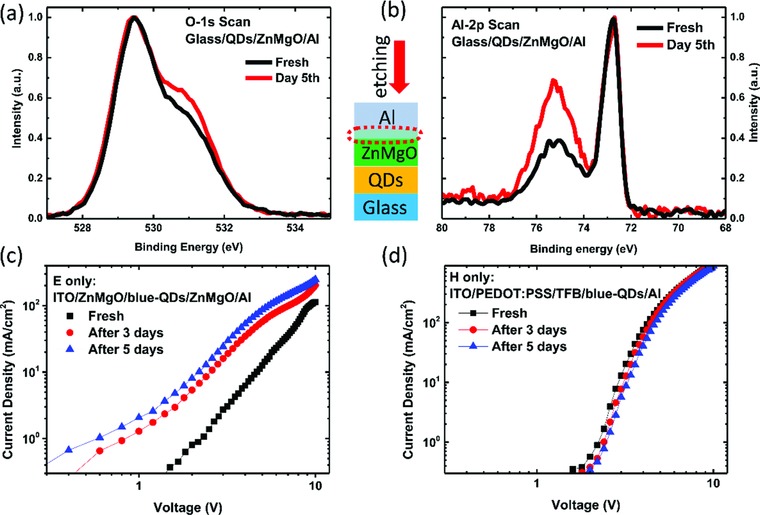
XPS spectra of a) O 1s scan and b) Al 2p scan at the interface of Al/ZnMgO. *J*–*V* characteristics of c) electron‐only device and d) hole‐only device tested at different time period.

We speculate that besides enhancing the electron injection, the interfacial AlO*_x_* can suppress the exciton quenching and thus improves the efficiency of the devices. To verify our hypothesis, the samples were characterized by PL and time‐resolved PL (TRPL) spectroscopy. To ensure the accuracy of the measurement, the PL intensity was collected using an integrating sphere and the excitation conditions were kept the same for different measurements. As shown in **Figure** **5**a and Figure S4 (Supporting Information), after 5 d aging, the PL intensities are slightly decreased for the samples quartz/QDs/ZnMgO, and the PL decay curves are almost the same, indicating that the aged QDs and ZnMgO do not contribute to efficiency improvement. However, for the samples with Al cathode: quartz/QDs/ZnMgO/Al, the PL intensities are significantly enhanced after 5 d aging, as shown in Figure 5b. The TRPL decay curves shown in Figure 5c were fitted with a triexponential function, and the average exciton lifetime is estimated by the relationship τ_*av*_ = *A*_1_τ_1_ + *A*_2_τ_2_ + *A*_3_τ_3_ with the parameters of time components (τ_*i*_) and corresponding weights (*A_i_*). The fitting results are summarized in Table S1 (Supporting Information). After 5 d aging, the exciton lifetimes of the blue, green, and red QDs are increased from 3.68, 4.87, and 9.12 ns to 4.30, 5.31, and 10.26 ns, respectively. The improvement of PL intensity and the lengthened exciton lifetime indicate that the exciton quenching is effectively suppressed, which is likely due to the formation of interfacial AlO*_x_*. As schematically illustrated in Figure 5d, the defect states originated from oxygen vacancies could act as charge recombination centers,^33^, ^34^ which trap holes from QDs and electrons from metal electrode. More specifically, the electrons from Al cathode can be effectively captured by the defect states of ZnMgO. Due to the well‐aligned energy levels, the trapped electrons can further transfer to the valence band of QDs and thus quench the excitons. The formation of interfacial AlO*_x_* can effectively block the pathway of electron trapping, and consequently suppress the exciton quenching, as briefly illustrated in Figure 5d. To conclude, the efficiency improvement of red and green QLEDs is mainly due to the suppression of exciton quenching, while both enhancement of electron injection and suppression of exciton quenching play the major role in enhancing the performance of blue QLEDs. This also explains why blue QLEDs exhibit the highest efficiency improvement of 2.6‐fold, as compared to 1.3‐ and 1.25‐fold of the green and the red devices, respectively.

**Figure 5:**
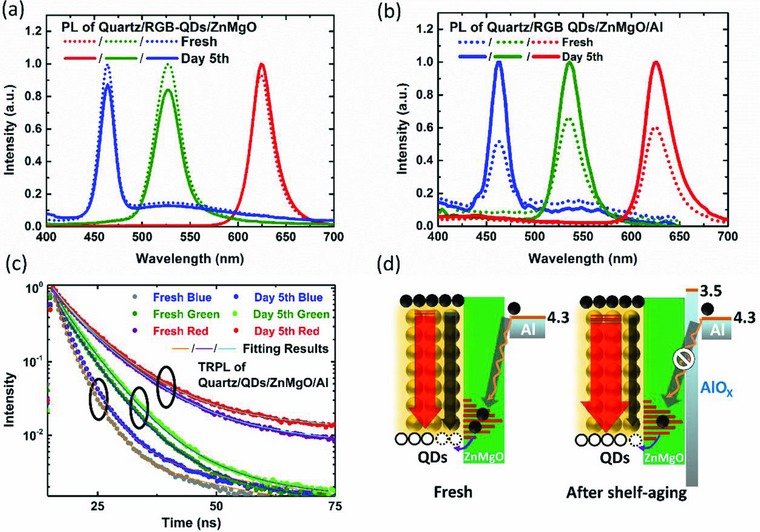
a,b) PL spectra and c) TRPL decay curves of blue, green, and red samples tested at different time period. d) Schematic illustration of exciton quenching process.

In conclusion, the origin of positive aging has been thoroughly investigated. We observed that the QLEDs exhibit higher efficiency and larger current density after shelf‐aging for several days. Through studying the effects of encapsulation resins, ETL materials, and cathode materials on the aging behavior, we identify that it is the interfacial reaction between Al and ZnMgO that accounts for the positive aging. The Al slowly reacts with the oxygen from ZnMgO, leading to the formation of AlO*_x_* and the accumulation of oxygen vacancies at the interface of Al/ZnMgO. The electron injection is effectively enhanced due to the formation of alloyed AlZnMgO interface and the improvement of ZnMgO conductivity. Moreover, the interfacial AlO*_x_* can effectively block the pathway of electron trapping and thus suppress the exciton quenching by metal electrode. Due to enhancement of electron injection and suppression of exciton quenching, the 8 d shelf‐aged blue, green, and red QLEDs exhibit a 2.6‐, 1.3‐, and 1.25‐fold efficiency improvement, respectively. Our studies disclose the origin of positive aging and provide a new insight into the exciton quenching mechanisms, which would be useful for further constructing efficient QLED devices.

## Experimental Section

*Device Fabrication*: QLEDs with structure of glass/ITO/PEDOT:PSS (40 nm)/TFB (30 nm)/QDs (R/G/B)/ZnMgO (35 nm)/Al (100 nm) were fabricated, where ITO, PEDOT:PSS (poly(3,4‐ethylenedioxythiophene) polystyrene sulfonate), TFB (Poly[(9,9‐dioctylfluorenyl‐2,7‐diyl)‐co‐(4,4′‐(*N*‐(*p*‐butylphenyl))diphenylamine)]), QDs, ZnMgO, and Al work as anode, hole injection layer (HIL), hole transport layer (HTL), light emission layer (EML), ETL, and cathode, respectively. Except for the thermally evaporated Al cathode, all other function layers were sequentially deposited onto glass/ITO substrates by spin‐coating with solutions using orthogonal solvents. To make the QLEDs, the ITO glasses (sheet resistance = 25 Ω sq^−1^) were firstly cleaned in ultrasonic detergent for 30 min, followed by soaking in ultrasonic deionized water for 10 min and baking in an oven for 30 min. Pior to deposition, the ITO substrates were treated by a UV‐ozone cleaner for 30 min. After UV‐ozone treatment, the PEDOT:PSS (Clevios AI 4083) hole injection layer was spin‐casted at 3000 rpm and baked at 130 °C for 20 min. Then the TFB (8 mg mL^−1^ in chlorobenzene) hole transport layer was coated at 3000 rpm for 40 s and annealed at 120 °C for 20 min. And then, the 15 mg mL^−1^ red‐QD (Cd*_x_*Zn_1‐_ *_x_*Se/ZnS/OT, core, and shell ≈12.2 nm, obtained from Mesolight Inc., quantum yield ≈85%), 10 mg mL^−1^ green‐QD (CdZnSeS/ZnS/oleic acid, core and shell ≈11.6 nm, obtained from Mesolight Inc., quantum yield ≈85%), and 10 mg mL^−1^ blue‐QDs (CdZnSeS/ZnS/OT, core and shell ≈10.5 nm, obtained from Mesolight Inc., quantum yield ≈85%) dissoved in octane solution were spin‐coated at 3000, 3000, and 2500 for 40 s, respectively. After that, the commercially available Zn_0.85_Mg_0.15_O nanoparticles were spin coated on QDs at 3000 rpm from a 20 mg mL^−1^ ethanol solution and baked at 100 °C for 10 min. Finally, a 100 nm Al cathode was evaporated in a high‐vacuum evaporation chamber at a base pressure of 5 × 10^−4^ Pa. After fabrication, the samples were kept in a N_2_‐filled glove box.

For inverted QLEDs with a structure of glass/ITO/ZnMgO (35 nm)/QDs (R/G/B)/TcTa (30 nm)/NPB (30 nm)/HATCN (10 nm)/Al, where TcTa = Tris(4‐carbazoyl‐9‐ylphenyl)amine, NPB = *N*,*N*′‐diphenyl‐*N*,*N*′‐bis(1‐naphthy) (1,1′‐biphenyl)‐4,4′diamine, HATCN = Dipyrazino[2,3‐f:2′,3′‐h]quinoxaline‐2,3,6,7,10, 11‐hexacarbonitrile. ZnMgO NPs were spin‐coated at 3000 rpm onto the precleaned substrate from a 20 mg mL^−1^ ethanol solution and baked at 120 °C for 10 min. After that, QDs dissoved in octane solution were spin‐coated and baked at 100 °C for 5 min. Subsequently, other functional layers were thermally evaporated in a vacuum evaporator with a base pressure of 5 × 10^−4^ Pa. After fabrication, the samples were kept in a N_2_‐filled glove box.

*Device Characterization*: The thicknesses of the solution‐processed films were measured by a Bruker DektakXT Stylus Profiler. The evaporation rates and the thicknesses of TcTa, NPB, HATCN, and Al were in situ monitored by a quartz crystal microbalance. The measurement results were further calibrated by a Bruker DektakXT Stylus Profiler. The PL and TRPL spectra were measured with Edinburgh instruments FS5 Fluorescence Spectrometer. The EL spectra and the current density−voltage−luminance (*J*−*V*−*L*) characteristics were measured using a Keithley 2614B programmable source meter and a PR670 spectrometer.

## Conflict of Interest

The authors declare no conflict of interest.

## Supporting information

Supplementary

## References

[1] Y. Yang , Y. Zheng , W. Cao , A. Titov , J. Hyvonen , J. R. Manders , J. Xue , P. H. Holloway , L. Qian , Nat. Photonics 2015, 9, 259.

[2] X. L. Dai , Y. Z. Deng , X. G. Peng , Y. Z. Jin , Adv. Mater. 2017, 29, 1607022.

[3] D. Bozyigit , V. Wood , MRS Bull. 2013, 38, 731.

[4] H. Shen , Q. Lin , W. Cao , C. Yang , N. T. Shewmon , H. Wang , J. Niu , L. S. Li , J. Xue , Nanoscale 2017, 9, 13583. 28876000 10.1039/c7nr04953f

[5] H. Zhang , X. Sun , S. Chen , Adv. Funct. Mater. 2017, 27, 1700610. 10.1002/adfm.201604872 PMC5513192 28729818

[6] C.‐Y. Han , H. Yang , J. Korean Ceram. Soc. 2017, 54, 449.

[7] V. L. Colvin , M. C. Schlamp , A. P. Alivisatos , Nature 1994, 370, 354.

[8] X. L. Dai , Z. X. Zhang , Y. Z. Jin , Y. Niu , H. J. Cao , X. Y. Liang , L. W. Chen , J. P. Wang , X. G. Peng , Nature 2014, 515, 96. 25363773 10.1038/nature13829

[9] H. Zhang , S. M. Chen , X. W. Sun , ACS Nano 2018, 12, 697. 29253334 10.1021/acsnano.7b07867

[10] Y. Z. Sun , Y. B. Jiang , H. R. Peng , J. L. Wei , S. D. Zhang , S. M. Chen , Nanoscale 2017, 9, 8962. 28548170 10.1039/c7nr02099f

[11] N. Koch , ChemPhysChem 2010, 8, 1438. 10.1002/cphc.200700177 17539032

[12] G. E. Jabbour , B. Kippelen , N. R. Armstrong , N. Peyghambarian , Appl. Phys. Lett. 1998, 73, 1185.

[13] F. So , J. Kido , P. Burrows , MRS Bull. 2008, 33, 663.

[14] M. A. El‐Sayed , Acc. Chem. Res. 2004, 37, 326. 15147173 10.1021/ar020204f

[15] G. Schwartz , M. Pfeiffer , S. Reineke , K. Walzer , K. Leo , Adv. Mater. 2007, 19, 3672.

[16] H. Zhang , N. Sui , X. C. Chi , Y. H. Wang , Q. H. Liu , H. Z. Zhang , W. Y. Ji , ACS Appl. Mater. Interfaces 2016, 8, 31385. 27781427 10.1021/acsami.6b09246

[17] W. K. Bae , L. A. Padilha , Y. S. Park , H. McDaniel , I. Robel , J. M. Pietryga , V. I. Klimov , ACS Nano 2013, 7, 3411. 23521208 10.1021/nn4002825

[18] Y. S. Park , W. K. Bae , J. M. Pietryga , V. I. Klimov , ACS Nano 2014, 8, 7288. 24909861 10.1021/nn5023473

[19] K. H. Lee , J. H. Lee , H. D. Kang , B. Park , Y. Kwon , H. Ko , C. Lee , J. Lee , H. Yang , ACS Nano 2014, 8, 4893. 24758609 10.1021/nn500852g

[20] K. P. Acharya , A. Titov , J. Hyvonen , C. G. Wang , J. Tokarz , P. H. Holloway , Nanoscale 2017, 9, 14451. 28926075 10.1039/c7nr05472f

[21] S. S. Xu , H. B. Shen , C. H. Zhou , H. Yuan , C. S. Liu , H. Z. Wang , L. Ma , L. S. Li , J. Phys. Chem. C 2011, 115, 20876.

[22] R. G. Xie , U. Kolb , J. X. Li , T. Basche , A. Mews , J. Am. Chem. Soc. 2005, 127, 7480. 15898798 10.1021/ja042939g

[23] S. Jun , E. Jang , Angew. Chem., Int. Ed. 2013, 52, 679. 10.1002/anie.201206333 23166006

[24] M. K. Choi , J. Yang , D. C. Kim , Z. H. Dai , J. Kim , H. Seung , V. S. Kale , S. J. Sung , C. R. Park , N. S. Lu , T. Hyeon , D. H. Kim , Adv. Mater. 2018, 30, 1704333. 10.1002/adma.201703279 29068560

[25] H. K. Kim , K. K. Kim , S. J. Park , T. Y. Seong , I. Adesida , J. Appl. Phys. 2003, 94, 4225.

[26] H. K. Kim , S. H. Han , T. Y. Seong , W. K. Choi , Appl. Phys. Lett. 2000, 77, 1647.

[27] H. Kim , K. K. Kim , S. N. Lee , J. H. Ryou , R. D. Dupuis , Appl. Phys. Lett. 2011, 98, 488.

[28] T. Cheng , Z. Wang , S. Jin , F. Wang , Y. Bai , H. Feng , B. You , Y. Li , T. Hayat , Z. Tan , Adv. Opt. Mater. 2017, 5, 1700035

[29] D. Abbaszadeh , G. A. H. Wetzelaer , N. Y. Doumon , P. W. M. Blom , J. Appl. Phys. 2016, 119, 539.

[30] B. J. Jin , S. H. Bae , S. Y. Lee , S. Im , Mater. Sci. Eng., B 2000, 71, 301.

[31] D. C. Look , D. C. Reynolds , J. R. Sizelove , R. L. Jones , C. W. Litton , G. Cantwell , W. C. Harsch , Solid State Commun. 1998, 105, 399.

[32] H. Peng , Y. Jiang , S. Chen , Nanoscale 2016, 8, 17765. 27714133 10.1039/c6nr05181b

[33] E. Polydorou , A. Zeniou , D. Tsikritzis , A. Soultati , I. Sakellis , S. Gardelis , T. A. Papadopoulos , J. Briscoe , L. C. Palilis , S. Kennou , E. Gogolides , P. Argitis , D. Davazoglou , M. Vasilopoulou , J. Mater. Chem. A 2016, 4, 11844.

[34] A. van Dijken , E. A. Meulenkamp , D. Vanmaekelbergh , A. Meijerink , J. Lumin. 2000, 90, 123.

