# Supplementary material for: Origin of Positive Aging in Quantum‐Dot Light‐Emitting Diodes
Source: Adv Sci (Weinh). 2018 Jul 3;5(10):1800549. doi: 10.1002/advs.201800549 (PMC6193172; doi:10.1002/advs.201800549)
Supplement: Supplementary file 1 — Supplementary [file ADVS-5-1800549-s001.pdf]

Copyright WILEY-VCH Verlag GmbH & Co. KGaA, 69469 Weinheim, Germany, 2018.

ADVANCED SCIENCE Open Access

Supporting Information

for *Adv. Sci.,* DOI: 10.1002/advs.201800549

Origin of Positive Aging in Quantum-Dot Light-Emitting Diodes

*Qiang Su, Yizhe Sun, Heng Zhang, and Shuming Chen**

Supporting Information

Origin of Positive Aging in Quantum-Dot Light-Emitting Diodes

Qiang Su, Yizhe Sun, Heng Zhang, Shuming Chen*

Department of Electrical and Electronic Engineering, Southern University of Science and Technology, Shenzhen, 518055, P. R. China

chen.sm@sustc.edu.cn

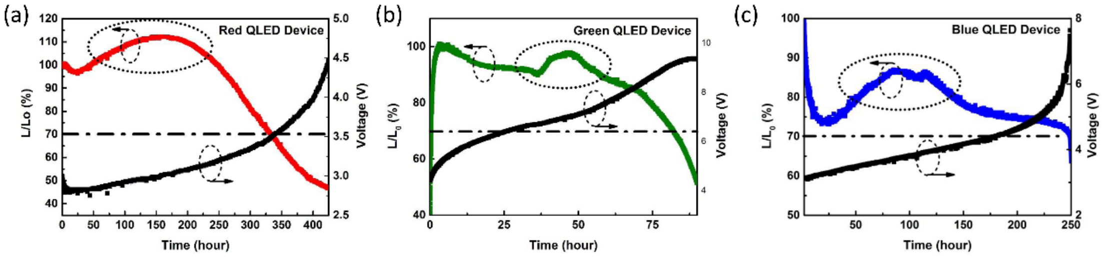

Figure S1. The lifetime of red/green/blue QLEDs with conventional structure.

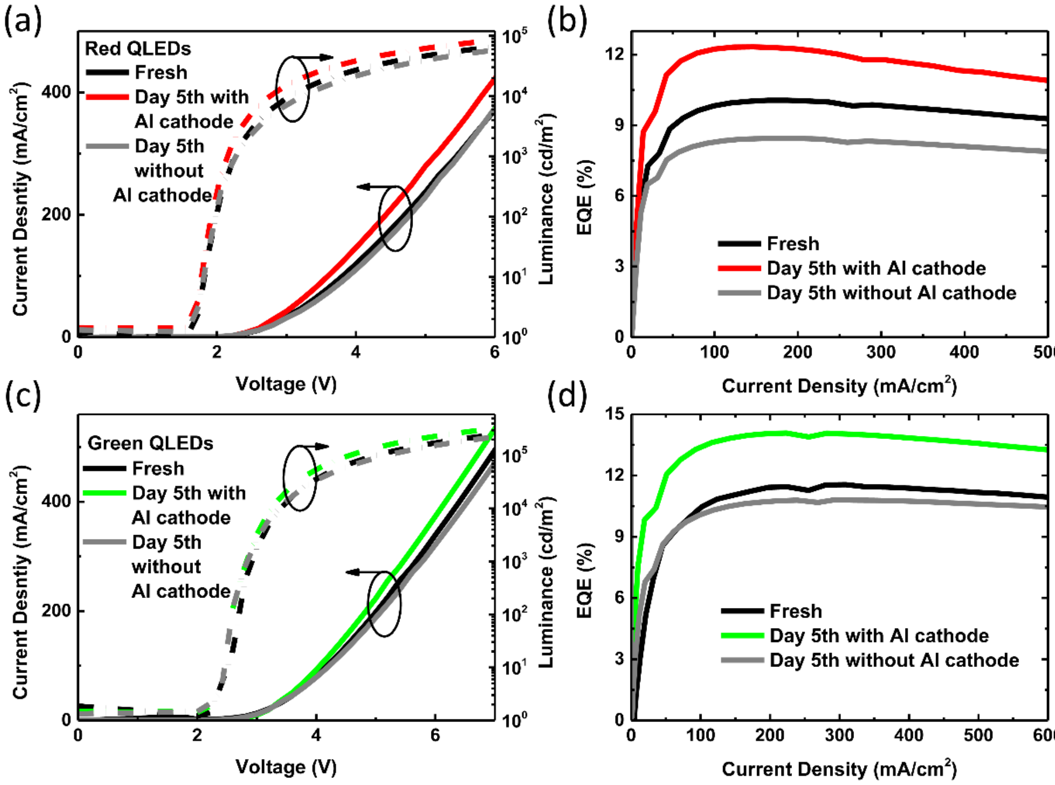

Figure S2. J-V-L characteristics and EQE-J characteristics of (a), (b) red QLEDs and (c), (d) green QLEDs tested at different time period with and without Al.

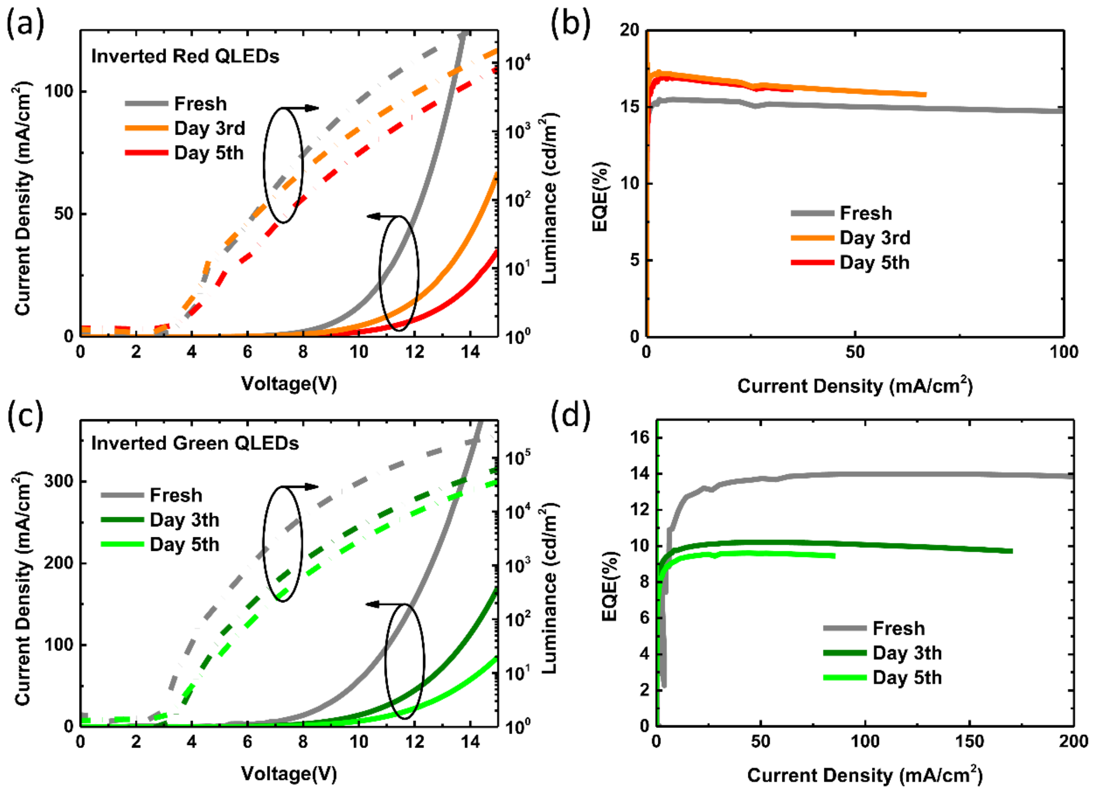

Figure S3. J-V-L characteristics and EQE-J characteristics of (a), (b) red QLEDs and (c), (d) green QLEDs tested at different time period with inverted structure.

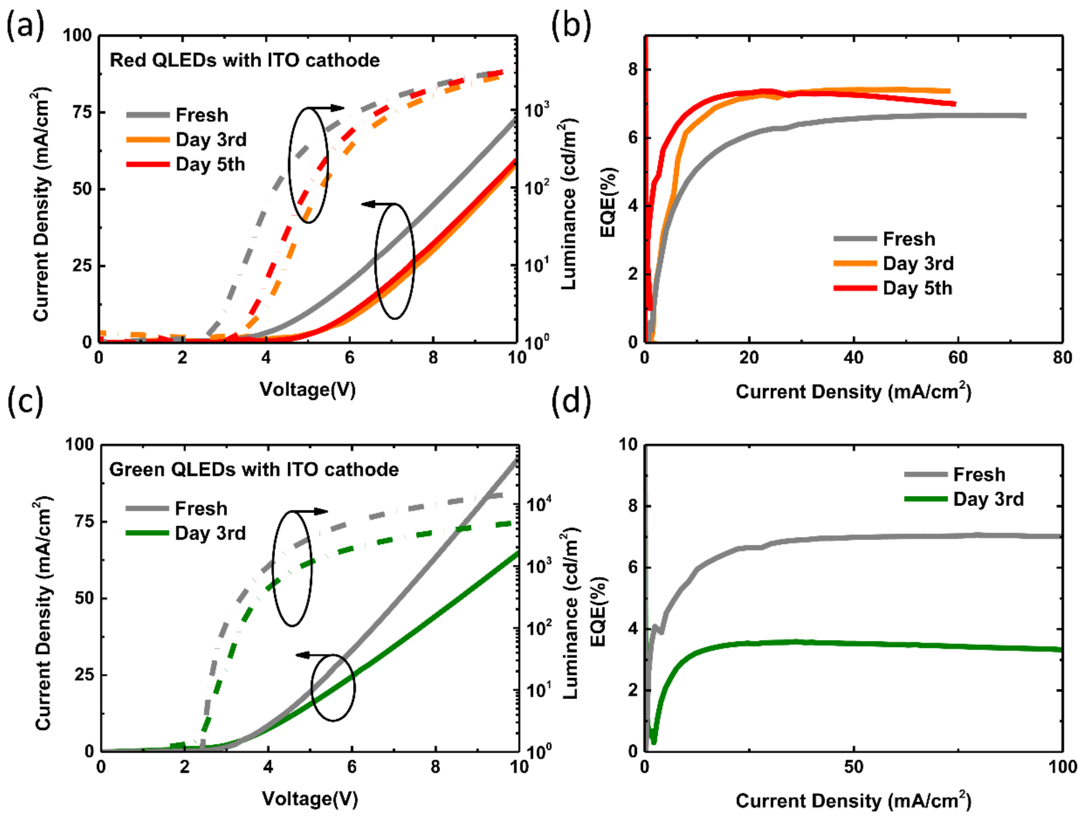

Figure S4. J-V-L characteristics and EQE-J characteristics of (a), (b) red QLEDs and (c), (d) green QLEDs tested at different time period with ITO cathode.

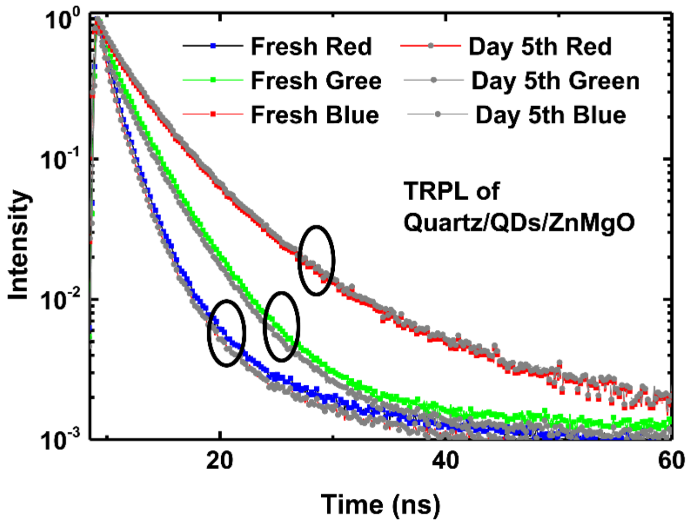

Figure S5. TRPL decay curves of blue, green and red samples tested at different time period with structure of quartz/QDs/ZnMgO.

Table S1. TRPL fitting results

**S2**	\	**τ_1 (ns)** **(A_i %)**	**τ_2 (ns)** **(A_i %)**	**τ_3 (ns)** **(A_i %)**	x^2	**τ_{av} (ns)**
**Blue**	Fresh	1.36(32.02)	3.38(62.26)	19.86(5.73)	1.290	3.68
	Day 5th	1.55(32.43)	3.78(61.02)	22.80(6.55)	1.295	4.30
**Green**	Fresh	0.97(8.10)	4.36(80.57)	11.26(11.33)	1.418	4.87
	Day 5th	1.27(7.21)	4.75(81.52)	11.92(11.27)	1.542	5.31
**Red**	Fresh	3.07(19.22)	7.72(69.92)	28.90(10.86)	1.456	9.12
	Day 5th	3.15(17.64)	7.92(70.58)	34.84(11.79)	1.642	10.26
